# Whole Genome Expression Analyses of miRNAs and mRNAs Suggest the Involvement of miR-320a and miR-155-3p and their Targeted Genes in Lithium Response in Bipolar Disorder

**DOI:** 10.3390/ijms20236040

**Published:** 2019-11-30

**Authors:** Claudia Pisanu, Eleni Merkouri Papadima, Carla Melis, Donatella Congiu, Annalisa Loizedda, Nicola Orrù, Stefano Calza, Sandro Orrù, Carlo Carcassi, Giovanni Severino, Raffaella Ardau, Caterina Chillotti, Maria Del Zompo, Alessio Squassina

**Affiliations:** 1 Department of Biomedical Science, Section of Neuroscience and Clinical Pharmacology, University of Cagliari, Monserrato, 09042 Cagliari, Italy; claudia.pisanu@unica.it (C.P.); e.merkouri.papadima@gmail.com (E.M.P.); calymelis@gmail.com (C.M.); dcongiu@unica.it (D.C.); severino@unica.it (G.S.); delzompo@unica.it (M.D.Z.); 2 Consiglio Nazionale delle Ricerche (C.N.R.), Istituto di Ricerca Genetica e Biomedica (I.R.G.B.), Monserrato, 09042 Cagliari, Italy; annalisa.loizedda@irgb.cnr.it; 3 Medical Genetics, R. Binaghi Hospital, ASSL Cagliari, ATS Sardegna, 09021 Cagliari, Italy; orru.nic@tiscali.it (N.O.); s.orru@unica.it (S.O.); carcassi@unica.it (C.C.); 4 Unit of Biostatistics and Bioinformatics, Department of Molecular and Translational Medicine, University of Brescia, 25121 Brescia, Italy; stefano.calza@unibs.it; 5 Big & Open Data Innovation Laboratory, University of Brescia, 25121 Brescia, Italy; 6 Medical Genetics, Department of Medical Sciences and Public Health, University of Cagliari, 09042 Cagliari, Italy; 7 Unit of Clinical Pharmacology of the University Hospital of Cagliari, 09042 Cagliari, Italy; Ardau.raf@tiscali.it (R.A.); katine@tiscali.it (C.C.)

**Keywords:** mood stabilizers, mood disorders, microRNA, miRNA, pharmacogenetics, epigenetics, next generation sequencing

## Abstract

Lithium is the mainstay in the maintenance of bipolar disorder (BD) and the most efficacious pharmacological treatment in suicide prevention. Nevertheless, its use is hampered by a high interindividual variability and important side effects. Genetic and epigenetic factors have been suggested to modulate lithium response, but findings so far have not allowed identifying molecular targets with predictive value. In this study we used next generation sequencing to measure genome-wide miRNA expression in lymphoblastoid cell lines from BD patients excellent responders (ER, *n* = 12) and non-responders (NR, *n* = 12) to lithium. These data were integrated with microarray genome-wide expression data to identify pairs of miRNA/mRNA inversely and significantly correlated. Significant pairs were prioritized based on strength of association and in-silico miRNA target prediction analyses to select candidates for validation with qRT-PCR. Thirty-one miRNAs were differentially expressed in ER vs. NR and inversely correlated with 418 genes differentially expressed between the two groups. A total of 331 of these correlations were also predicted by in-silico algorithms. miR-320a and miR-155-3p, as well as three of their targeted genes (*CAPNS1* (Calpain Small Subunit 1) and *RGS16* (Regulator of G Protein Signaling 16) for miR-320, *SP4* (Sp4 Transcription Factor) for miR-155-3p) were validated. These miRNAs and mRNAs were previously implicated in psychiatric disorders (miR-320a and *SP4*), key processes of the central nervous system (*CAPNS1*, *RGS16*, *SP4*) or pathways involved in mental illnesses (miR-155-3p). Using an integrated approach, we identified miRNAs and their targeted genes potentially involved in lithium response in BD.

## 1. Introduction

Bipolar disorder (BD) is a disabling psychiatric disorder characterized by the recurrence of depressive and manic/hypomanic episodes [1]. Being associated with premature mortality, disability, and high risk of suicide, BD exerts a significant socioeconomic burden [2]. After more than 60 years of use, the mood stabilizer lithium is still a first line treatment for BD, being effective in reducing recurrences and suicide risk [3,4]. As a maintenance treatment, lithium is highly effective in at least 30% of chronically treated patients with a complete remission of symptoms. However, there is general consensus that lithium has a certain degree of effectiveness in about 60% of patients, while it is ineffective in about 1/3 [5,6]. Additionally, loss of efficacy can be observed after discontinuation in patients previously showing a good response to the drug [7]. Lithium has also been shown to protect against mood switching [8]. A number of clinical predictors of lithium response have been described and discussed over the years. Features like an episodic pattern of mania–depression intervals, absence of rapid cycling, low rates of comorbid conditions, high age at illness onset, absence of family history for schizophrenia, and family history for lithium response have been associated with better lithium response [9,10,11,12,13]. On the other hand, poor lithium response has been associated with rapid-cycling, mood incongruent psychosis, and BD type II (BD-II) [11]. Nevertheless, clinical predictors have shown poor sensitivity and specificity in most of the studies, and reliable tools for a better management of lithium treatment, especially to predict response, are still missing. 

These features have stimulated intensive research to identify genetic and molecular predictors of response and disentangle its complex biological mechanisms [14,15]. The most important effort in this sense is being made by the International Consortium on Lithium Genetics (ConLiGen), which has recently published the largest genome-wide association study (GWAS) on lithium response [16]. Results from this study suggested the involvement of two long non-coding RNAs in lithium response. The implication of the non-coding portion of the genome in psychiatric phenotypes and response to psychotropic medications has been suggested by an increasing number of studies [17]. The largest evidence so far has been reported for microRNAs (miRNAs), which are involved in the regulation of several physiological functions, including cell differentiation, development and homeostasis, via modulation of expression of messenger RNAs (mRNA) as well as of other non-coding RNAs [18]. Interestingly, several studies have shown that lithium interferes with the expression of a number of miRNA and their targeted genes [19,20], suggesting they could play a role in modulating lithium’s clinical efficacy. To date, only two studies investigated genome-wide miRNAs in lithium response using two distinct approaches and study design [21,22], but none of these investigations suggested the involvement of specific miRNA/mRNA pairs in either the mechanism of action of lithium or in its clinical efficacy.

In this study, we aimed at identifying pairs of miRNAs-mRNAs involved in lithium response. To this purpose, we sequenced small non-coding RNAs with next generation sequencing (NGS) in lymphoblastoid cell lines (LCLs) from BD patients characterized for lithium response, and integrated these data with genome-wide messenger RNA (mRNA) expression levels from the same subjects. The use of NGS allowed us a more extensive and sensitive evaluation of miRNAs compared to other approaches. The miRNA-mRNA pairs identified by our study could help shedding light on the complex genetic and epigenetic architecture of lithium response, ultimately leading to the identification of potential peripheral biomarkers and new drug targets. 

## 2. Results

### 2.1. Genome-Wide Analysis of miRNAs and mRNAs

**Figure 1:**
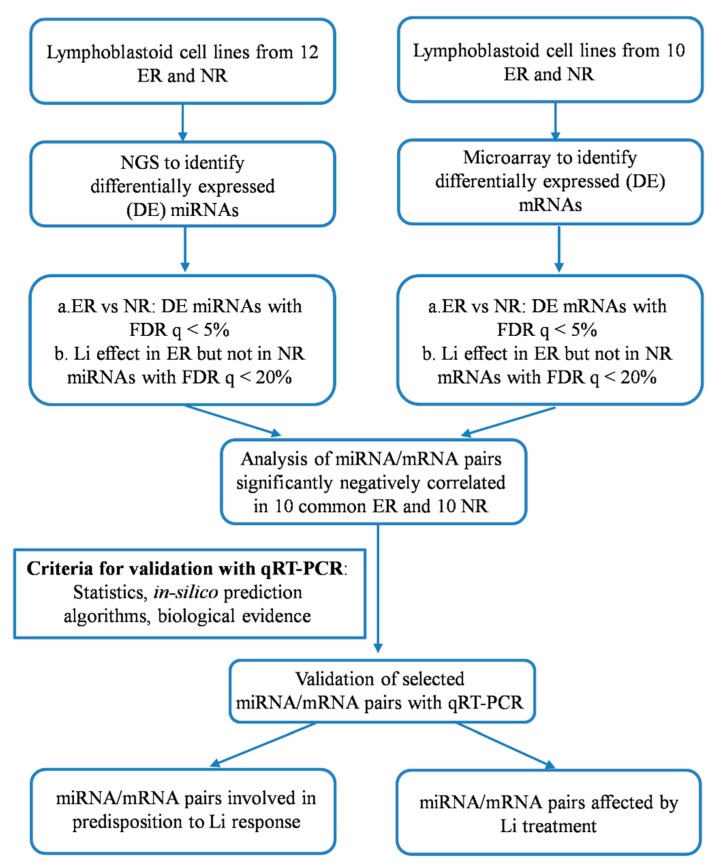
Workflow of the study. Abbreviations: DE, differentially expressed; ER, excellent responders; FDR, false discovery rate; HG, human genome, LCL, lymphoblastoid cell lines; Li, lithium; mRNA, messenger RNA, miRNA, microRNA; NGS, next generation sequencing; NR, non-responders; qRT-PCR, quantitative reverse transcription-PCR.

A workflow of the study is reported in Figure 1. 

At base calling, 96.4% of the bases passed the Phred Quality Score threshold (Q > 30). Preprocessing and alignment of the reads resulted in 998 annotated miRNAs out of approximately 2500 reference miRNAs. A total of 194 miRNAs had at least two counts per million (CPM) of reads in at least one group. Among these, 52 were significantly differentially expressed between excellent responders (ER) and non-responders (NR) with a false discovery rate (FDR) <0.05 (Table 1), while 5 were significantly differentially expressed after in vitro lithium treatment exclusively in ER with an FDR <0.2 (Table S1). 

In the microarray dataset, as previously reported [23], a total of 2060 mRNAs were differentially expressed between ER and NR at FDR <0.05. Additionally, 56 mRNAs were differentially expressed after in vitro lithium treatment exclusively in ER at FDR <0.2. 

### 2.2. Correlation between miRNAs and mRNAs Expression Levels

Among 44 miRNAs with at least two CPM of reads in all samples and 2060 mRNAs, both significantly differentially expressed between ER and NR, we identified 513 significant negative correlations (including 31 miRNAs and 418 unique mRNAs, FDR *q* < 0.05). Among these, 331 correlations (including 30 miRNAs and 277 genes) were also predicted by at least one of the tested in-silico algorithms (Table S2). The network of miRNA–mRNA pairs is represented in Figure S1. The network shows two main clusters of down-regulated and up-regulated miRNAs, centered around miR-320a and miR-155-3p, respectively.

Among 5 miRNAs and 56 mRNAs significantly differentially expressed after lithium treatment exclusively in ER, no correlation was significant after correction for multiple testing. We identified 15 nominally significant negative correlations (including five miRNAs and eleven unique mRNAs, *p* < 0.05), seven of which (including five miRNAs and six genes) were also predicted by at least one in-silico algorithm (Table S3).

### 2.3. Validation of Selected miRNAs-mRNAs Pairs with qRT-PCR 

**Figure 2:**
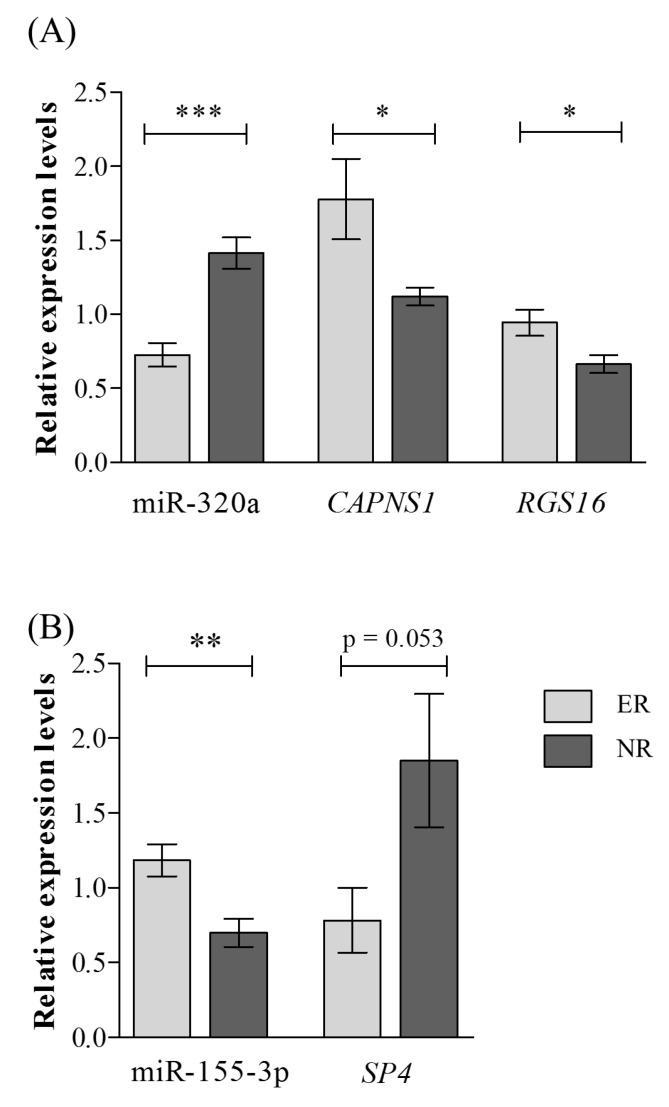
Results from qRT-PCR for miRNAs and target genes differentially expressed between lithium excellent responders and non-responders. Results from qRT-PCR for (**A**) miR-320 and its targets *CAPNS1* and *RGS16* and (**B**) miR-155-3p and its target *SP4*. Abbreviations: ER, excellent responders; qRT-PCR, quantitative reverse transcription-PCR; NR, non-responders. *** *p* < 0.0001; ** *p* = 0.002, * *p* < 0.05.

Among the prioritized pairs of miRNA/mRNA differentially expressed in ER compared to NR, we selected three miRNAs (hsa-miR-155, hsa-miR-138 and hsa-miR-320) and seven mRNAs (four genes targeted by miR-320a: *RGS16*, *CAPNS1*, *BHLHE40,* and *RHOA*, and three targeted by miR-155-3p: *SP4*, *AUTS2,* and *KYAT1*) (Figures S2 and S3). Additionally, we selected one miRNA (hsa-miR-27a) affected by in vitro lithium treatment and negatively correlated with at least one target with an unadjusted *p* < 0.05.

hsa-miR-320a was confirmed to be significantly down-regulated (fold change (FC) = 0.51, *p* < 0.0001)) and hsa-miR-155-3p up-regulated (FC: 1.70, *p* = 0.003) in ER (Table 2 and Figure 2). Two of the four selected hsa-miR-320a targets were validated: *CAPNS1* (FC = 1.59, *p* = 0.040) and *RGS16* (FC = 1.41, *p* = 0.017), which were significantly up-regulated in ER (Table 2 and Figure 2). The hsa-miR-155-3p target *SP4* showed a trend for down-regulation (FC = 0.43, *p* = 0.053).

## 3. Discussion

This study represents the first NGS genome-wide investigation of miRNAs in BD patients characterized for lithium response. We integrated these data with genome-wide mRNA expression levels from the same subjects to identify pairs of miRNAs and target genes differentially expressed in lithium ER compared to NR. Our results suggest that miR-320a, miR-155-3p and their target mRNAs might constitute relevant players in modulating clinical response to lithium. Lithium ER showed increased levels of miR-320a and reduced levels of its targets *CAPNS1* and *RGS16* compared to NR. MiR-320a is located on chromosome 8 and was previously suggested to be involved in major depressive disorder (MDD) [24] and schizophrenia (SCZ) [25]. Specifically, a recent study investigating plasmatic levels of seven miRNAs previously implicated in psychiatric disorders found miR-320a to be down-regulated in patients with MDD (*n* = 50) compared to healthy controls (*n* = 41) [24]. MiR-320a was also shown to be up-regulated in the neuroblastoma SH-SY5Y cell line after 24 h of in vitro treatment with the antidepressant fluoxetine 10-μM, although this result was not validated by subsequent experiments from the same group [26].

A potential involvement of miRNAs of the miR-320 family was also suggested for other psychiatric disorders. MiR-320d was shown to be up-regulated in the plasma of depressed patients in a sample including 16 patients with MDD and 14 controls [27]. A recent study showed that miR-320a-3p and miR-320b were down-regulated in serum from patients with SCZ without treatment (*n* = 3) compared to patients under treatment (*n* = 3) and healthy controls (*n* = 3) [25]. This finding was confirmed by the same authors in a larger cohort including 59 antipsychotic-naïve SCZ patients and 60 controls [25]. Genes targeted by miR-320a and validated in our study have been previously suggested to play a crucial role in a range of mechanisms, including neuronal survival and differentiation, apoptosis, and synaptic plasticity. Specifically, *CAPNS1* encodes a regulatory subunit essential for the stability and function of calpains, i.e., a family of calcium-dependent cysteine proteinases involved in several functions, including synaptic plasticity and neuroprotection, dendritic branching complexity and spine density [28]. The regulatory subunit encoded by *CAPNS1* is common to the two major calpain isoforms in the brain, i.e., calpain-1 and calpain-2, which play opposite roles in synaptic plasticity and neurodegeneration: while calpain-1 has been suggested to play a neuroprotective role, calpain-2 activation is involved in neurodegeneration and reduced synaptic plasticity [29]. Previous studies have shown that *CAPNS1* was underexpressed [30] and hypermethylated [31] in the prefrontal cortex of patients with SCZ compared to healthy controls. However, no studies to date suggested a potential role of this gene in mood disorders or in response to mood stabilizers. We observed increased expression levels of *CAPNS1* in patients with excellent response to lithium compared to NR. Being the protein encoded by this gene essential for the function of both calpain isoforms, further studies will be needed to understand how increased levels of this gene might be involved in modulating lithium response in BD. Similarly, we found increased levels of *RGS16* in lithium ER. This miR-320a target encodes a member of the regulator of G protein signaling family. Specifically, *RGS16* plays a crucial role in the circadian regulation of cyclic AMP (cAMP) in the suprachiasmatic nucleus [32]. This brain region, located in the ventral part of the anterior hypothalamus, coordinates and synchronizes the daily rhythms of sleep and wakefulness as well as circadian rhythms of a wide range of homeostatic functions [32]. Intriguingly, a large body of evidence supports an involvement of circadian rhythm disturbances in BD. Patients with BD often show alterations in the sleep–wake rhythm, eveningness chronotype, and abnormal melatonin secretion [33]. Furthermore, circadian rhythm dysfunctions have been suggested to predict relapses in BD patients [34]. In a recent study, skin fibroblasts from BD patients responders to lithium treatment were more likely to show a short circadian period, a linear relationship between period and phase, and period shortening effects of lithium compared to non-responders [34]. In accordance with this hypothesis, several studies provided evidence of contribution of genetic variation or differential expression of circadian genes to the pathophysiology of BD or in the predisposition to lithium response [35,36,37,38,39,40,41]. To our knowledge, our study suggests for the first time the involvement of *RGS16* in response to lithium treatment. 

In our study we also showed an overexpression of miR-155-3p in LCLs from ER to lithium treatment compared to NR. Interestingly, a previous study had shown miR-155 to be upregulated at day 4 and day 16 in LCLs from 10 BD patients and 10 siblings after in vitro treatment with lithium 1mM [19]. MiR-155 plays an important role in inflammatory response. This miRNA modulates differentiation and activation of cells playing a crucial role in innate and adaptive immune systems [42]. However, contrasting findings have been reported as regard to the role of miR-155, with some studies suggesting it to be part of a negative feedback loop which downregulates synthesis of inflammatory cytokines [43] and other reports suggesting a putative pro-inflammatory role [42]. *SP4*, one of miR-155-3p targets showing statistical significance in our study, codifies for a zinc finger transcription factor highly expressed in neurons and previously suggested to be involved in BD and SCZ [44]. Interestingly, acute in vitro treatment with lithium was found to stabilize SP4 protein via a reduction of its phosphorilation at serine 770 in cultured rat cerebellar granule neurons [45] Accordingly, the authors showed a significantly reduced phosphorilated SP4 (pSP4)/SP4 ratio in peripheral blood mononuclear cells from first-episode psychosis patients treated with lithium (*n* = 6) compared to patients treated with other drugs (*n* = 8) [45]. Unfortunately, in this study, no information on lithium response was available. Our observation of reduced *SP4* expression levels in lithium ER might suggest that reduced phosphorylation and consequent stabilization of the SP4 protein previously reported could be associated with a negative feedback on *SP4* gene expression via up-regulation of miR-155. 

To date, only two studies have investigated the role of miRNAs in lithium response using a hypothesis-free approach [21,22]. The study published by Hunsberger and colleagues (2015) explored differences in the expression of miRNAs and mRNAs induced by lithium treatment in vitro on LCLs from BD patients characterized for lithium response [21]. The genome-wide expression of miRNAs and mRNA was carried out using the microarray technology, and data were integrated using an integrative genomic tool called GRANITE (Genetic Regulatory Analysis of Networks Investigational Tool Environment). The findings showed that the let-7 family of miRNAs was down-regulated by lithium in both responders and NR, but none of the mRNAs selected for validation was significant. The second study published by Reinbold and colleagues in 2018, analyzed the association between lithium response and miRNA-based statistics using genotyping data for single nucleotide polymorphisms (SNP) within and in proximity of miRNA loci in the ConLiGen dataset [22]. While a number of SNPs showed a nominal association with lithium response, no association survived correction for multiple testing. 

Our results have to be interpreted in light of some limitations. Firstly, we investigated peripheral changes in expression of miRNAs and mRNAs using LCLs, which might not reflect brain changes. Despite being widely used for research purposes in the neuropsychiatric field, LCLs present some criticisms that are still under debate and that specifically concern the effect of immortalization on the host genome. On the other hand, LCLs present several advantages, as they are easy to set, can be grown in standardized and controlled conditions thus reducing variability, and can be used to test the effect of in vitro assays on molecular targets [46]. Another limit of our study is represented by the limited sample size, which might have not allowed us to identify miRNA/mRNA pairs with small effect sizes. This was in part overcome by the use of a sample of patients selected at the extreme ends of the scale to evaluate clinical response to lithium, an approach that allows reducing the phenotypic heterogeneity within the same response group and increasing the diversity among different response groups. The main strength of our study is the use of NGS to measure genome-wide expression of miRNAs and the integration of these data with mRNAs. While we also integrated information gathered from the algorithms available online for miRNAs target prediction, our discovery was primarily based on the negative correlations between miRNAs and mRNA measured on the same sample set. This allowed to merge experimental evidence with the predictive information, thus providing more robust support for the identified miRNA-mRNA pairs. On the other hand, we did not perform functional validation of the miRNA-mRNA interactions, and as such, our findings require further investigation.

In conclusion, results from our study suggest that miR-320, miR-155 and their targeted genes might be involved in modulating lithium response in BD, thus providing more support on the involvement of the non-coding portion of the genome in response to psychotropic medications. The miRNAs and genes identified in our work might be the object of further studies aimed at exploring the predictive value of these molecular targets using a prospective, longitudinal study design.

## 4. Materials and Methods

### 4.1. Sample

The study was carried out in a sample of patients retrospectively evaluated for lithium response and selected from an existing cohort of 350 patients recruited in the last 20 years at the Lithium Clinic of the Clinical Psychopharmacology Centre of the University Hospital of Cagliari, Italy. Patients were diagnosed according to Research Diagnostic Criteria (RDC) [47] and DSM-IV criteria, using personal semi-structured interviews (Schedule for Affective Disorder and Schizophrenia Lifetime Version (SADS-L)) [48] and a systematic review of their medical records. Exclusion criteria included comorbidities with any disorder of the DSM-IV axes I and II. Clinical response to maintenance treatment with lithium was evaluated using the “Retrospective Criteria of Long-Term Treatment Response in Research Subjects with Bipolar Disorder” (Alda scale) [49,50], as previously described [23]. Briefly, the scale measures the degree of improvement in the course of treatment (Criterion A) weighted against clinical factors considered relevant for determining whether or not the observed improvement is due to the treatment (Criteria B1–B5). The degree of response for each patient is quantified with a score from 0 to 10 (total score), obtained by subtracting the score B from the score A. Patients with total score (TS) equal to 7 or higher are considered lithium responders, while patients with a TS equal or lower than 2 are considered NR. For this study, we selected patients at the two extreme ends of the scale, in order to increase the within-subjects homogeneity in terms of response definition. The sample for NGS included 12 ER (male/female = 5/7, mean age at sampling, years ± standard deviation (SD) = 45.2 ± 16.1 TS ≥ 8) and 12 NR (male/female = 5/7, mean age at sampling, years ± SD = 44.5 ± 13.0 TS = 0). The genome-wide gene expression study included a subset of this sample, specifically 10 ER (male/female = 5/5, mean age at sampling, years ± SD = 45.3 ± 16.1, TS ≥ 8) and 10 NR (male/female = 4/6, mean age at sampling, years ± SD = 44.5 ± 14.1, TS = 0) as previously described [23]. The research protocol followed the principles of the Declaration of Helsinki and was approved by the Ethics Committee of the University of Cagliari, Italy (approval number: 348/FC/2013, approved in date: 21 June 2013). All participants signed informed written consent after a detailed description of the study procedures.

### 4.2. LCLs and In Vitro Lithium Treatment

LCLs were already available for the entire cohort and had generated following standard procedures. Briefly, Epstein–Barr virus immortalized LCLs were established from lymphocytes and stored in liquid nitrogen at the time of enrollment in previous molecular studies [23]. For the present study, LCLs from selected patients (and with passage numbers <5) were thawed and cultured using a standard protocol previously described [51]. Once LCLs reached the required cell count (6–9 × 10^6^ cells), two equivalent aliquots were transferred into separate flasks. One aliquot was cultured with medium supplemented with 1 mM lithium chloride (LiCl), while the other one was cultured with drug-free medium, under identical conditions. After 7 days of treatment, cells were harvested for total RNA isolation. 

### 4.3. Genome-Wide NGS Analysis of miRNAs

Total RNA was extracted using the miRNeasy Mini Kit (QIAGEN GmbH-Hilden, Hilden, Germany). Quantification and quality evaluation of miRNAs was performed using the Small RNA Analysis Kit (DNF-470) adjusted for the Fragment Analyzer™ Automated CE System (Advanced Analytical Technologies, Ankeny, IA, USA) and the Qubit RNA BR (Broad-Range) Assay Kit for Qubit 2.0 Fluorometer (Invitrogen, Carlsbad, CA, USA). During library preparation, small RNAs having the 3′ hydroxyl end and the 5′-phosphate group produced by Dicer were selectively ligated to adapters, retrotranscribed, amplified and indexed, and finally purified to produce pure miRNA libraries. Subsequently, 1 µg of total RNA with TruSeq Small RNA Library Prep Kit (Illumina, San Diego, CA, US) was processed according to the Illumina^®^ TruSeq^®^ Small RNA Library Prep protocol for miRNAs. The amplified cDNA constructs were purified using the AMPure XP beads (Beckman Coulter, Brea, CA, US). The Small RNA Libraries were subsequently validated using the High Sensitivity Small DNA Fragment Analysis Kit (DNF-477) and loaded on Fragment Analyzer™ Automated CE System (Advanced Analytical Technologies). Qubit^®^ 2.0 Fluorometer (Thermo Fisher Scientific, Waltham, MA, USA) was used to check the purity and concentration of the samples. Sequencing was performed on a MiSeq^®^ System instrument (Illumina). The instrument was loaded with MiSeq^®^ Reagent Kit v3 and the samples were loaded on standard flow cells. 

### 4.4. Genome-Wide Microarray Analysis of mRNAs

Microarrays experiments were performed for a previous study [23] on LCLs from a sample of 10 patients also included in the NGS study. Briefly, RNA was extracted using TRI reagent solution (Ambion, Austin, TX, USA). The quality of RNA was considered to be adequate when the A260/A280 ratio assessed using a NanoDrop ND-1000 spectrophotometer was in the range of 1.8–2.0, and the RNA integrity number assessed using the Agilent 2100 Bioanalyzer (Agilent, Santa Clara, CA, USA) was in the range of 7–10. First, 100 ng of total RNA were amplified and used to generate a sense-strand complementary DNA (cDNA) with incorporated 2’-Deoxyuridine 5’-Triphosphate (dUTP), using the Ambion WT Expression Kit (Applied Biosystems, Foster City, CA, USA). In a second step, cDNA fragmentation and labeling were carried out using the Affymetrix GeneChip WT Terminal Labeling Kit. Samples were hybridized to GeneChip Human Gene ST 1.0 arrays (Affymetrix, Santa Clara, CA, USA). The arrays were placed in the hybridization oven for 17 h at 45 °C, and then washed and stained in the GeneChip Fluidics Station 450 (Affymetrix). Finally, the arrays were scanned using the GeneChip Scanner 3000 7G AutoLoader (Affymetrix). 

### 4.5. Validation with Quantitative Reverse Transcription-PCR (qRT-PCR)

Pairs of miRNAs and mRNAs for validation with qRT-PCR were selected according to the following criteria: (1) Both the miRNAs and the mRNAs were differentially expressed between ER and NR or significantly altered by in vitro lithium treatment exclusively in ER; (2) significant inverse correlation between miRNA and mRNA expression levels in our dataset; (3) at least one in-silico database predicting the expression correlation; (4) biological evidence from the literature supporting a role of the miRNA or the mRNA in biological processes related to neuronal or brain functions or neuropsychiatric disorders. 

Selected miRNAs were measured using TaqMan^®^ Small RNA Assays (Applied Biosystems,). cDNAs were synthesized using the TaqMan^®^ MicroRNA Reverse Transcription Kit (Applied Biosystems) and specific stem-looped primers included in the TaqMan^®^ Small RNA Assay, following the manufacturer’s indications. The following qRT-PCR reactions were run in triplicate in a StepOnePlus™ instrument (Applied Biosystems) using TaqMan™ MicroRNA Assays (hsa-miR-320: 002277; hsa-miR-155: 002287; hsa-miR-138; 002284; hsa-miR-27a: 000408). RNA, U6 Small Nuclear 6, Pseudogene (RNU6B) was used as an endogenous control. A pooling of all samples as a calibrator and no-template controls (NTC) were included in each plate.

Selected mRNAs were measured using mRNA targets TaqMan^®^ Assays (Applied Biosystems). cDNAs were synthesized using the high capacity cDNA reverse transcription kit (Life Technologies Corporation, Carlsbad, CA, USA) according to the manufacturer’s protocol. TaqMan^®^ Gene Expression Master Mix (Applied Biosystems) was mixed together with probes and primers for the selected targets (Sp4 Transcription Factor (*SP4*): Hs00162095_m1; Basic Helix-Loop-Helix Family Member E40 (*BHLHE40*): Hs00186419_m1; Calpain Small Subunit 1 (*CAPNS1*): Hs00998426_m1; Ras Homolog Family Member A (*RHOA*): Hs01051295_m1; Regulator Of G Protein Signaling 16 (*RGS16*): Hs00892674_m1; Activator Of Transcription And Developmental Regulator AUTS2 (*AUTS2*): Hs01688766_m1; Kynurenine Aminotransferase 1 (*KYAT1*): Hs00187858). Glyceraldehyde-3-Phosphate Dehydrogenase (*GAPDH*) was used as an endogenous control. A pooling of all samples as a calibrator and NTCs were included in each plate. 

### 4.6. Data Analysis

#### 4.6.1. Genome-Wide Analysis NGS of miRNAs 

Data quality control was initially performed with the instrument software using Phred Quality Scores, Q scores, filtering for clusters with Q > 30. FASTQ reads were preprocessed before alignment for adapter removal using cutadapt [52] and shortened to a max length of 28 bp. FASTQ file reads were then aligned to a reference of mature miRNAs using bowtie [53]. Subsequently, miRNAs were filtered keeping only those with at least 2 CPM of reads in all samples within at least one experimental group (ER vs. NR, lithium treated vs. untreated cells in ER or in NR). Data were normalized based on effective library size and computation of dispersion. Differential expression was computed using a negative binomial model using edgeR [54] in R [55]. Correction for multiple testing was conducted according to the Benjamini–Hochberg FDR (BH) procedure. In the analysis comparing ER vs. NR, an FDR *q* threshold of 5% was used. In the analyses evaluating the effect of in vitro lithium treatment of miRNA expression levels, significance was defined based on a less stringent FDR threshold of 20% to increase sensitivity. This choice was based on findings from our previous work [23] suggesting that LiCl in vitro has a small-medium effect on gene expression modifications in LCLs and as such, by using an FDR threshold of 5%, we would have likely excluded the majority of genes significantly altered by lithium treatment. We created two lists of miRNAs affected by lithium in ER and NR, respectively, and selected those altered by lithium exclusively in ER, as these miRNAs are more likely to be involved in modulating clinical efficacy of lithium.

#### 4.6.2. Genome-Wide Analysis of mRNAs 

Analyses of the microarray dataset were previously described [23]. Briefly, geneChip data quality control was performed using Expression Console Software (Affymetrix). Transcriptome data were normalized using the Robust Multi-array Average algorithm and duplicated or missing Entrez IDs were removed. Genes were tested for differential expression between ER and NR, as well as before and after in vitro lithium treatment using the linear models implemented in limma [56]. As in the case of the NGS data, we used two FDR thresholds of 5% and 20% to identify mRNAs differentially expressed between ER and NR or affected by in vitro lithium treatment exclusively in ER, respectively. 

#### 4.6.3. Correlation between miRNA and mRNA Expression Levels

In the subsample of 10 ER and 10 NR for which both miRNA and mRNA expression levels were available, correlations between log2-transformed expression levels of miRNAs and mRNAs significantly differentially expressed between ER and NR, or affected by in vitro lithium treatment exclusively in ER, were analyzed using miRComb v0.9.1 [57] in R. This package allows combining genome-wide miRNA and mRNA expression data to select negative correlations and obtain a list of miRNA-mRNA interactions adjusted for multiple testing. Negative correlations were considered significant in case of an FDR < 5%. For this analysis, miRNAs were filtered keeping only those with at least 2 CPM of reads in all samples within the experimental groups in which the correlation analysis was conducted (ER vs. NR and lithium treated vs. untreated cells in ER). Significant negative correlations were further prioritized based on *in silico* predicted interactions using MiRWalk version 2.0 [58]. MiRWalk documents miRNA binding sites within the sequence of a gene as well as binding sites resulting from different miRNA-target prediction algorithms. We used seven different prediction algorithms (MiRWalk, MicroT v4, MiRanda, miRDB, RNA22, RNAhybrid and TargetScan). The network of miRNA-mRNAs pairs significantly and negatively correlated in our data, as well as predicted by at least one algorithm, was represented using Cytoscape version 3.7.1 [59] using a force-directed layout algorithm, which places nodes with high connectivity at the center of the graph. Pairs of miRNAs and mRNAs were selected for validation with qRT-PCR according to the criteria reported in Section 4.5.

#### 4.6.4. Validation with qRT-PCR

For both miRNAs and mRNAs, relative expression levels were measured with the comparative *C*_t_ method (ΔΔ*C*_t_) using RNU6B or GAPDH as the endogenous controls, respectively. FCs of the difference between ER and NR, or between treated and not treated cells were calculated with the 2^−ΔΔ*C*t^ equation. Differences between ER and NR were analyzed using Student *t*-tests, while differences between baseline and lithium-treated samples were analyzed using the paired samples *t*-test. A *p*-value < 0.05 was considered significant. Analyses were conducted using SPSS version 22 (SPSS, Inc., Chicago, IL, USA) and GraphPad Prism version 8.00 (GraphPad Software, San Diego, CA, USA).

## Figures and Tables

**Table 1:** Significantly differentially expressed miRNAs between lithium excellent responders and non-responders at FDR threshold *q* < 0.05.

miRNA	FC	*p*	FDR *q*
hsa-miR-320a	0.55	3.2 × 10^−10^	3.8 × 10^−8^
hsa-miR-125a-5p	0.16	6.6 × 10^−8^	3.9 × 10^−6^
hsa-miR-148a-3p	2.23	1.2 × 10^−7^	4.9 × 10^−6^
hsa-miR-574-3p	0.32	5.4 × 10^−7^	1.6 × 10^−5^
hsa-miR-1273h-3p	0.49	3.5 × 10^−5^	0.0008
hsa-miR-22-3p	1.79	7.2 × 10^−5^	0.0014
hsa-miR-9-5p	0.57	0.0001	0.0019
hsa-miR-26b-5p	1.78	0.0001	0.0019
hsa-miR-378a-5p	0.43	0.0002	0.0030
hsa-miR-223-3p	4.13	0.0003	0.0030
hsa-miR-155-3p	2.27	0.0003	0.0031
hsa-miR-505-3p	0.50	0.0005	0.0043
hsa-miR-744-5p	1.59	0.0005	0.0043
hsa-let-7e-5p	0.32	0.0005	0.0043
hsa-miR-138-5p	0.32	0.0006	0.0044
hsa-miR-181a-3p	2.61	0.0006	0.0044
hsa-miR-15a-5p	1.62	0.0006	0.0044
hsa-miR-941	0.54	0.0007	0.0045
hsa-miR-148b-3p	2.32	0.0007	0.0045
hsa-miR-652-3p	0.46	0.0008	0.0049
hsa-miR-130b-3p	0.69	0.0009	0.0050
hsa-miR-15b-3p	1.81	0.0013	0.0068
hsa-miR-345-5p	0.56	0.0014	0.0070
hsa-miR-454-5p	2.14	0.0019	0.0096
hsa-miR-4677-3p	2.51	0.0021	0.0102
hsa-miR-374a-3p	1.74	0.0024	0.0111
hsa-miR-19b-3p	1.59	0.0029	0.0125
hsa-let-7d-3p	0.71	0.0030	0.0125
hsa-miR-181d-5p	0.55	0.0034	0.0136
hsa-miR-101-3p	1.76	0.0035	0.0136
hsa-miR-629-5p	0.46	0.0036	0.0136
hsa-miR-574-5p	0.49	0.0039	0.0144
hsa-miR-378a-3p	0.68	0.0042	0.0150
hsa-miR-148a-5p	1.61	0.0044	0.0152
hsa-miR-142-3p	1.47	0.0045	0.0152
hsa-miR-454-3p	1.45	0.0050	0.0163
hsa-miR-142-5p	1.58	0.0051	0.0163
hsa-miR-598-3p	0.51	0.0052	0.0163
hsa-let-7f-5p	1.33	0.0054	0.0163
hsa-miR-27a-5p	1.66	0.0084	0.0241
hsa-let-7a-5p	1.29	0.0085	0.0241
hsa-miR-210-5p	1.41	0.0087	0.0241
hsa-miR-30e-3p	1.43	0.0087	0.0241
hsa-miR-146a-5p	1.95	0.0117	0.0315
hsa-miR-23a-3p	0.73	0.0131	0.0346
hsa-miR-15b-5p	1.39	0.0149	0.0385
hsa-miR-425-5p	0.73	0.0171	0.0430
hsa-miR-197-3p	0.71	0.0182	0.0450
hsa-miR-335-3p	3.38	0.0204	0.0489
hsa-miR-421	1.34	0.0208	0.0489
hsa-miR-26a-5p	1.34	0.0210	0.0489
hsa-miR-194-5p	0.60	0.0215	0.0490

Abbreviations: FC, fold change; FDR, false discovery rate.

**Table 2:** Findings from NGS and microarray analyses and validation with qRT-PCR of the selected miRNA/mRNA pairs differentially expressed between lithium excellent responders and non-responders.

	NGS/Microarray			qRT-PCR	
	FC	*p*	FDR *q*	FC	*p*
**miRNA**					
**hsa-miR-320a**	0.55	3.2 × 10^−10^	3.8 × 10^−8^	**0.51**	**3.2 × 10^−5^**
**hsa-miR-155-3p**	2.27	0.0003	0.0031	**1.70**	**0.003**
hsa-mir-138	0.32	0.0006	0.0044	0.63	0.180
**mRNA**					
***CAPNS1***	1.21	9.2 × 10^−5^	0.0021	**1.59**	**0.040**
***RGS16***	1.52	1.3 × 10^−5^	0.0006	**1.42**	**0.017**
*BHLHE40*	1.37	2.4 × 10^−6^	0.0002	1.05	0.167
*RHOA*	1.11	3.1 × 10^−5^	0.0010	1.60	0.556
*SP4*	0.68	2.9 × 10^−5^	0.0001	0.43	0.053
*KYAT1*	0.75	1.5 × 10^−8^	7.2 × 10^−6^	0.43	0.065
*AUTS2*	0.43	8.5 × 10^−5^	0.0020	1.71	0.250

miRNA and genes measured with NGS and microarray, respectively, and validated with qRT-PCR are indicated in bold. Abbreviations: FC, fold change; FDR, false discovery rate; NGS, next generation sequencing; qRT-PCR, quantitative reverse transcription-PCR.

## Supplementary Materials

The following are available online at https://www.mdpi.com/1422-0067/20/23/6040/s1.

## Abbreviations

AUTS2	Activator of Transcription and Developmental Regulator
BD	Bipolar disorder
cAMP	Cyclic AMP
CAPNS1	Calpain Small Subunit 1
BH	Benjamini–Hochberg
BHLHE40	Basic Helix-Loop-Helix Family Member E40
cDNA	Complementary DNA
ConLiGen	Consortium on Lithium Genetics
DE	Differentially expressed
CPM	Counts per million
dUTP	2’-Deoxyuridine 5’-Triphosphate
ER	Excellent responders
FC	Fold change
FDR	False discovery rate
GAPDH	Glyceraldehyde-3-Phosphate Dehydrogenase
GWAS	Genome-wide association study
KYAT1	Kynurenine Aminotransferase 1
LCL	Lymphoblastoid cell lines
LiCl	Lithium chloride
MDD	Major depressive disorder
mRNA	Messenger RNA
miRNA	microRNA
NGS	Next generation sequencing
NR	Non-responders
NTC	No-template controls
qRT-PCR	Quantitative reverse transcription-PCR
RDC	Research Diagnostic Criteria
RHOA	Ras Homolog Family Member A
RNU6B	RNA, U6 Small Nuclear 6, Pseudogene
RGS16	Regulator of G Protein Signaling 16
SCZ	Schizophrenia
SNP	Single nucleotide polymorphisms
SP4	Sp4 Transcription Factor
SADS-L	Schedule for Affective Disorder and Schizophrenia Lifetime Version
SD	Standard deviation
SP4	Sp4 Transcription Factor
TS	Total score
