# Supplementary material for: Whole Genome Expression Analyses of miRNAs and mRNAs Suggest the Involvement of miR-320a and miR-155-3p and their Targeted Genes in Lithium Response in Bipolar Disorder
Source: Int J Mol Sci. 2019 Nov 30;20(23):6040. doi: 10.3390/ijms20236040 (PMC6928759; doi:10.3390/ijms20236040)
Supplement: Supplementary file 1 [file ijms-20-06040-s001.pdf]

International Journal of Molecular Sciences

MDPI

Table S1. List of miRNAs differentially expressed after *in vitro* lithium treatment exclusively in excellent responders at an FDR $q < 0.2$.

miRNA	FC	p	FDR q
hsa-miR-29b-3p	1.41	0.0010	0.10
hsa-miR-374a-5p	1.54	0.0025	0.13
hsa-miR-27a-3p	1.18	0.0040	0.14
hsa-miR-23a-3p	1.25	0.0058	0.15
hsa-miR-106b-5p	1.28	0.0086	0.18

Abbreviations: FC, fold change; FDR, false discovery rate.

Table S2. Pairs of miRNAs and mRNAs differentially expressed between lithium excellent responders and non-responders, significantly negatively correlated and predicted by in-silico algorithms. The significant threshold was set at FDR $q < 0.05$.

miRNA	FC	mRNA	FC	Pearson's r	p	FDR q	*In-silico* score
hsa-miR-421	1.34	*PLCXD1*	0.74	-0.87	3.58×10^{-7}	0.0148	1
hsa-miR-155-3p	2.27	*RP2*	0.73	-0.84	1.53×10^{-6}	0.0218	1
hsa-miR-320a	0.55	*BHLHE40*	1.37	-0.84	2.00×10^{-6}	0.0218	6
hsa-miR-1273h-3p	0.49	*PEX14*	1.08	-0.84	2.14×10^{-6}	0.0218	1
hsa-miR-320a	0.55	*LSP1*	1.48	-0.83	2.36×10^{-6}	0.0218	1
hsa-miR-345-5p	0.56	*STRA6*	1.21	-0.83	3.17×10^{-6}	0.0235	1
hsa-miR-138-5p	0.32	*GABARAP*	1.14	-0.82	4.02×10^{-6}	0.0245	2
hsa-miR-138-5p	0.32	*ZBTB45*	1.16	-0.82	4.79×10^{-6}	0.0245	2
hsa-miR-320a	0.55	*FSTL3*	1.64	-0.82	5.51×10^{-6}	0.0245	1
hsa-miR-320a	0.55	*C9*	1.13	-0.81	6.10×10^{-6}	0.0245	2
hsa-miR-155-3p	2.27	*DNASE1L3*	0.29	-0.81	6.19×10^{-6}	0.0245	1
hsa-miR-9-5p	0.57	*PARP4*	1.13	-0.81	6.62×10^{-6}	0.0245	1
hsa-miR-15a-5p	1.62	*SMARCA2*	0.74	-0.80	1.01×10^{-5}	0.0304	1
hsa-miR-155-3p	2.27	*SP4*	0.68	-0.80	1.09×10^{-5}	0.0304	4
hsa-miR-378a-3p	0.68	*ATF7*	1.14	-0.80	1.15×10^{-5}	0.0304	2
hsa-miR-155-3p	2.27	*ZC3HC1*	0.88	-0.80	1.19×10^{-5}	0.0304	1
hsa-miR-421	1.34	*SFRP1*	0.51	-0.80	1.38×10^{-5}	0.0304	2
hsa-miR-374a-3p	1.74	*ENGASE*	0.84	-0.80	1.39×10^{-5}	0.0304	1
hsa-miR-574-3p	0.32	*TCEB3*	1.25	-0.80	1.39×10^{-5}	0.0304	2
hsa-miR-374a-3p	1.74	*PTPMT1*	0.91	-0.79	1.42×10^{-5}	0.0304	1
hsa-miR-1273h-3p	0.49	*ISCU*	1.19	-0.79	1.48×10^{-5}	0.0304	3
hsa-miR-345-5p	0.56	*RER1*	1.11	-0.79	1.57×10^{-5}	0.0304	2
hsa-miR-1273h-3p	0.49	*EDF1*	1.13	-0.79	1.58×10^{-5}	0.0304	2
hsa-miR-378a-3p	0.68	*DCAF11*	1.17	-0.79	1.64×10^{-5}	0.0304	1
hsa-miR-320a	0.55	*PAPSS2*	2.59	-0.79	1.75×10^{-5}	0.0314	1
hsa-miR-138-5p	0.32	*BCL2L1*	1.39	-0.79	1.78×10^{-5}	0.0314	4
hsa-miR-374a-3p	1.74	*PLXNA1*	0.76	-0.79	1.90×10^{-5}	0.0321	1

hsa-miR-320a	0.55	*RHOA*	1.11	-0.79	1.99×10^{-5}	0.0321	1
hsa-miR-27a-5p	1.66	*DNAJB4*	0.73	-0.78	2.07×10^{-5}	0.0321	1
hsa-miR-345-5p	0.56	*CYB5D2*	1.24	-0.78	2.31×10^{-5}	0.0332	3
hsa-miR-378a-3p	0.68	*SIRT2*	1.14	-0.78	2.38×10^{-5}	0.0332	1
hsa-miR-378a-3p	0.68	*PPP1CA*	1.15	-0.78	2.69×10^{-5}	0.0338	2
hsa-miR-378a-3p	0.68	*ASCC1*	1.24	-0.78	2.86×10^{-5}	0.0349	1
hsa-miR-320a	0.55	*FAM3D*	1.09	-0.78	2.89×10^{-5}	0.0349	1
hsa-miR-574-3p	0.32	*RNF31*	1.17	-0.78	2.94×10^{-5}	0.0349	1
hsa-miR-574-3p	0.32	*CREM*	1.34	-0.78	2.97×10^{-5}	0.0349	1
hsa-miR-454-5p	2.14	*SIRT4*	0.88	-0.77	3.38×10^{-5}	0.0374	1
hsa-miR-155-3p	2.27	*BICD2*	0.89	-0.77	3.40×10^{-5}	0.0374	4
hsa-miR-125a-5p	0.16	*STS*	1.86	-0.77	3.52×10^{-5}	0.0374	4
hsa-miR-421	1.34	*ANGPTL2*	0.77	-0.77	3.59×10^{-5}	0.0374	4
hsa-miR-15a-5p	1.62	*DIABLO*	0.90	-0.77	3.69×10^{-5}	0.0374	1
hsa-miR-1273h-3p	0.49	*BOD1*	1.11	-0.77	3.73×10^{-5}	0.0374	1
hsa-miR-345-5p	0.56	*TSPYL1*	1.21	-0.77	3.75×10^{-5}	0.0374	1
hsa-miR-142-3p	1.47	*BDH1*	0.88	-0.77	3.85×10^{-5}	0.0374	2
hsa-miR-378a-3p	0.68	*DUSP4*	1.50	-0.77	3.91×10^{-5}	0.0374	5
hsa-miR-320a	0.55	*CA5B*	1.42	-0.77	3.98×10^{-5}	0.0374	3
hsa-miR-1273h-3p	0.49	*FPGS*	1.10	-0.77	4.04×10^{-5}	0.0374	2
hsa-miR-130b-3p	0.69	*GFOD2*	1.12	-0.77	4.24×10^{-5}	0.0374	1
hsa-miR-15a-5p	1.62	*WDR48*	0.82	-0.76	4.30×10^{-5}	0.0374	3
hsa-miR-1273h-3p	0.49	*MAPK8IP3*	1.10	-0.76	4.31×10^{-5}	0.0374	2
hsa-miR-941	0.54	*MFNG*	1.28	-0.76	4.35×10^{-5}	0.0374	2
hsa-miR-194-5p	0.6	*ST8SIA2*	1.14	-0.76	4.36×10^{-5}	0.0374	1
hsa-miR-9-5p	0.57	*IL2RA*	2.46	-0.76	4.38×10^{-5}	0.0374	2
hsa-miR-320a	0.55	*ADD1*	1.15	-0.76	4.49×10^{-5}	0.0374	4
hsa-miR-378a-3p	0.68	*ZNF70*	1.22	-0.76	4.52×10^{-5}	0.0374	3
hsa-miR-155-3p	2.27	*KYAT1*	0.75	-0.76	4.53×10^{-5}	0.0374	1
hsa-miR-374a-3p	1.74	*PIR*	0.73	-0.76	4.56×10^{-5}	0.0374	2
hsa-miR-378a-3p	0.68	*SH2B3*	1.31	-0.76	4.79×10^{-5}	0.0374	2
hsa-miR-505-3p	0.5	*VAPB*	1.14	-0.76	4.80×10^{-5}	0.0374	4
hsa-miR-320a	0.55	*EDEM1*	1.25	-0.76	4.83×10^{-5}	0.0374	5
hsa-miR-378a-3p	0.68	*ZBTB45*	1.16	-0.76	4.89×10^{-5}	0.0374	1
hsa-miR-148a-3p	2.23	*EIF2AK2*	0.84	-0.76	5.12×10^{-5}	0.0377	2
hsa-miR-574-3p	0.32	*PSMC1*	1.17	-0.76	5.22×10^{-5}	0.0377	1
hsa-miR-320a	0.55	*BCL2L1*	1.39	-0.76	5.25×10^{-5}	0.0377	1
hsa-miR-345-5p	0.56	*GRHL3*	1.18	-0.76	5.27×10^{-5}	0.0377	1
hsa-miR-374a-3p	1.74	*TNS3*	0.73	-0.76	5.34×10^{-5}	0.0377	1
hsa-miR-378a-3p	0.68	*ARPC4*	1.18	-0.76	5.46×10^{-5}	0.0382	2
hsa-miR-15a-5p	1.62	*CHMP7*	0.81	-0.76	5.56×10^{-5}	0.0385	1
hsa-miR-15a-5p	1.62	*VPRBP*	0.85	-0.76	5.64×10^{-5}	0.0385	1
hsa-miR-378a-3p	0.68	*TMEM8A*	1.23	-0.76	5.66×10^{-5}	0.0385	1

hsa-miR-181a-3p	2.61	*CD59*	0.84	-0.75	6.05×10^{-5}	0.0395	1
hsa-miR-378a-3p	0.68	*SCAMP2*	1.34	-0.75	6.12×10^{-5}	0.0395	1
hsa-miR-1273h-3p	0.49	*UPP1*	1.45	-0.75	6.16×10^{-5}	0.0395	1
hsa-miR-345-5p	0.56	*PLEKHG5*	1.08	-0.75	6.23×10^{-5}	0.0395	1
hsa-miR-1273h-3p	0.49	*CCM2*	1.17	-0.75	6.24×10^{-5}	0.0395	1
hsa-miR-378a-3p	0.68	*RPS6KA1*	1.19	-0.75	6.27×10^{-5}	0.0395	4
hsa-miR-1273h-3p	0.49	*TRAF4*	1.26	-0.75	6.27×10^{-5}	0.0395	2
hsa-miR-155-3p	2.27	*KPTN*	0.73	-0.75	6.31×10^{-5}	0.0395	1
hsa-miR-1273h-3p	0.49	*PDZD2*	1.36	-0.75	6.47×10^{-5}	0.0395	1
hsa-miR-155-3p	2.27	*NDRG3*	0.73	-0.75	6.67×10^{-5}	0.0395	6
hsa-miR-378a-3p	0.68	*DAP*	1.17	-0.75	6.74×10^{-5}	0.0395	2
hsa-miR-155-3p	2.27	*PHF16*	0.42	-0.75	6.77×10^{-5}	0.0395	4
hsa-miR-320a	0.55	*STAT5A*	1.25	-0.75	6.80×10^{-5}	0.0395	2
hsa-miR-505-3p	0.5	*CIRBP*	1.37	-0.75	6.87×10^{-5}	0.0395	1
hsa-miR-9-5p	0.57	*CD40LG*	1.14	-0.75	6.98×10^{-5}	0.0395	2
hsa-miR-574-3p	0.32	*ISG20L2*	1.34	-0.75	7.05×10^{-5}	0.0395	1
hsa-miR-378a-3p	0.68	*PIGT*	1.13	-0.75	7.05×10^{-5}	0.0395	3
hsa-miR-505-3p	0.5	*EDF1*	1.13	-0.75	7.06×10^{-5}	0.0395	1
hsa-miR-320a	0.55	*PEX11B*	1.15	-0.75	7.09×10^{-5}	0.0395	2
hsa-miR-320a	0.55	*RGS16*	1.52	-0.75	7.25×10^{-5}	0.0395	1
hsa-miR-320a	0.55	*PBLD*	1.17	-0.75	7.26×10^{-5}	0.0395	2
hsa-miR-320a	0.55	*GPR132*	1.50	-0.75	7.29×10^{-5}	0.0395	2
hsa-miR-155-3p	2.27	*HBP1*	0.80	-0.75	7.29×10^{-5}	0.0395	2
hsa-miR-9-5p	0.57	*HSD11B1*	1.18	-0.75	7.42×10^{-5}	0.0395	1
hsa-miR-320a	0.55	*ABR*	1.23	-0.75	7.47×10^{-5}	0.0395	3
hsa-miR-574-3p	0.32	*UFD1L*	1.18	-0.75	7.56×10^{-5}	0.0395	2
hsa-miR-155-3p	2.27	*AUTS2*	0.43	-0.75	7.69×10^{-5}	0.0399	1
hsa-miR-194-5p	0.6	*TSPYL1*	1.21	-0.75	7.75×10^{-5}	0.0399	2
hsa-miR-155-3p	2.27	*METTL3*	0.85	-0.75	7.82×10^{-5}	0.0399	1
hsa-miR-155-3p	2.27	*RNF168*	0.73	-0.75	7.93×10^{-5}	0.0399	1
hsa-miR-148a-3p	2.23	*TRIM5*	0.76	-0.75	8.05×10^{-5}	0.0399	3
hsa-miR-15a-5p	1.62	*SUV420H1*	0.87	-0.74	8.58×10^{-5}	0.0408	3
hsa-miR-27a-5p	1.66	*TRIM5*	0.76	-0.74	9.00×10^{-5}	0.0409	2
hsa-miR-1273h-3p	0.49	*GHDC*	1.11	-0.74	9.35×10^{-5}	0.0411	4
hsa-miR-454-5p	2.14	*KIF6*	0.75	-0.74	9.36×10^{-5}	0.0411	2
hsa-miR-378a-3p	0.68	*SLC9A8*	1.38	-0.74	9.74×10^{-5}	0.0418	3
hsa-miR-9-5p	0.57	*SEL1L*	1.22	-0.74	9.83×10^{-5}	0.0419	3
hsa-miR-345-5p	0.56	*ZNF335*	1.12	-0.74	9.90×10^{-5}	0.0419	1
hsa-miR-194-5p	0.6	*RGS16*	1.52	-0.74	9.94×10^{-5}	0.0419	2
hsa-miR-155-3p	2.27	*TBRG4*	0.86	-0.74	1.01×10^{-4}	0.0423	1
hsa-miR-155-3p	2.27	*CHEK2*	0.71	-0.74	1.02×10^{-4}	0.0423	1
hsa-miR-138-5p	0.32	*HSF2BP*	1.16	-0.74	1.03×10^{-4}	0.0423	1
hsa-miR-320a	0.55	*CAPNS1*	1.21	-0.74	1.04×10^{-4}	0.0425	3

hsa-miR-1273h-3p	0.49	*YY1AP1*	1.12	-0.74	1.08 x 10^{-4}	0.0427	2
hsa-miR-320a	0.55	*ASCC1*	1.24	-0.74	1.09 x 10^{-4}	0.0427	4
hsa-miR-378a-3p	0.68	*INPP5B*	1.25	-0.74	1.11 x 10^{-4}	0.0427	2
hsa-miR-320a	0.55	*INPP5B*	1.25	-0.74	1.11 x 10^{-4}	0.0427	2
hsa-miR-142-5p	1.58	*MRPS26*	0.92	-0.74	1.11 x 10^{-4}	0.0427	2
hsa-miR-574-3p	0.32	*BCL2L1*	1.39	-0.73	1.13 x 10^{-4}	0.0427	1
hsa-miR-138-5p	0.32	*POM121*	1.17	-0.73	1.14 x 10^{-4}	0.0427	3
hsa-miR-320a	0.55	*PIGL*	1.59	-0.73	1.14 x 10^{-4}	0.0427	1
hsa-miR-320a	0.55	*HSF2BP*	1.16	-0.73	1.14 x 10^{-4}	0.0427	1
hsa-miR-155-3p	2.27	*HLTF*	0.73	-0.73	1.15 x 10^{-4}	0.0427	2
hsa-miR-320a	0.55	*GGA3*	1.15	-0.73	1.16 x 10^{-4}	0.0427	6
hsa-miR-1273h-3p	0.49	*PFDN5*	1.15	-0.73	1.16 x 10^{-4}	0.0427	1
hsa-miR-374a-3p	1.74	*SLC35A1*	0.78	-0.73	1.16 x 10^{-4}	0.0427	1
hsa-miR-1273h-3p	0.49	*TRAPPC4*	1.32	-0.73	1.19 x 10^{-4}	0.0427	1
hsa-miR-15a-5p	1.62	*TGIF2*	0.88	-0.73	1.19 x 10^{-4}	0.0427	6
hsa-miR-941	0.54	*SLC35A2*	1.12	-0.73	1.22 x 10^{-4}	0.0427	2
hsa-miR-378a-3p	0.68	*PNOC*	1.40	-0.73	1.23 x 10^{-4}	0.0427	1
hsa-miR-574-3p	0.32	*RELL2*	1.13	-0.73	1.23 x 10^{-4}	0.0427	2
hsa-miR-320a	0.55	*GPATCH8*	1.13	-0.73	1.23 x 10^{-4}	0.0427	5
hsa-miR-9-5p	0.57	*EDEM1*	1.25	-0.73	1.24 x 10^{-4}	0.0427	5
hsa-miR-194-5p	0.6	*GRHL3*	1.18	-0.73	1.25 x 10^{-4}	0.0427	4
hsa-miR-320a	0.55	*KRT34*	1.16	-0.73	1.25 x 10^{-4}	0.0427	1
hsa-miR-155-3p	2.27	*TSHZ1*	0.72	-0.73	1.26 x 10^{-4}	0.0427	5
hsa-miR-505-3p	0.5	*H1F0*	1.40	-0.73	1.26 x 10^{-4}	0.0427	1
hsa-miR-125a-5p	0.16	*ACSL4*	1.22	-0.73	1.28 x 10^{-4}	0.0427	1
hsa-miR-320a	0.55	*CYB5D2*	1.24	-0.73	1.29 x 10^{-4}	0.0427	6
hsa-miR-941	0.54	*SETD6*	1.15	-0.73	1.32 x 10^{-4}	0.0427	2
hsa-miR-574-3p	0.32	*LRP10*	1.29	-0.73	1.33 x 10^{-4}	0.0427	1
hsa-miR-378a-3p	0.68	*LILRB4*	1.74	-0.73	1.33 x 10^{-4}	0.0427	2
hsa-miR-26a-5p	1.34	*FKBP10*	0.80	-0.73	1.33 x 10^{-4}	0.0427	1
hsa-miR-142-3p	1.47	*RGMB*	0.79	-0.73	1.34 x 10^{-4}	0.0427	3
hsa-miR-378a-3p	0.68	*SELPLG*	1.43	-0.73	1.35 x 10^{-4}	0.0427	2
hsa-miR-378a-3p	0.68	*PARP15*	1.30	-0.73	1.35 x 10^{-4}	0.0427	2
hsa-miR-378a-3p	0.68	*PIK3CG*	2.52	-0.73	1.36 x 10^{-4}	0.0427	5
hsa-miR-9-5p	0.57	*TMEM131*	1.40	-0.73	1.36 x 10^{-4}	0.0427	2
hsa-miR-378a-3p	0.68	*PAPSS2*	2.59	-0.73	1.38 x 10^{-4}	0.0427	2
hsa-miR-130b-3p	0.69	*RHOA*	1.11	-0.73	1.38 x 10^{-4}	0.0427	3
hsa-miR-320a	0.55	*ASB2*	4.49	-0.73	1.39 x 10^{-4}	0.0427	3
hsa-miR-155-3p	2.27	*EIF4EBP1*	0.80	-0.73	1.39 x 10^{-4}	0.0427	1
hsa-miR-9-5p	0.57	*DCAF11*	1.17	-0.73	1.40 x 10^{-4}	0.0427	2
hsa-miR-320a	0.55	*SDF4*	1.11	-0.73	1.41 x 10^{-4}	0.0427	1
hsa-miR-15a-5p	1.62	*SMAD4*	0.91	-0.73	1.42 x 10^{-4}	0.0427	6
hsa-miR-1273h-3p	0.49	*QPCTL*	1.33	-0.73	1.44 x 10^{-4}	0.0428	1

hsa-miR-155-3p	2.27	*PTOV1*	0.89	-0.73	1.45 x 10^{-4}	0.0428	1
hsa-miR-574-3p	0.32	*CASP7*	1.18	-0.73	1.46 x 10^{-4}	0.0428	2
hsa-miR-378a-3p	0.68	*INHBA*	1.29	-0.73	1.46 x 10^{-4}	0.0428	3
hsa-miR-155-3p	2.27	*CHI3L1*	0.32	-0.73	1.46 x 10^{-4}	0.0428	1
hsa-miR-138-5p	0.32	*DERL2*	1.18	-0.72	1.50 x 10^{-4}	0.0428	1
hsa-miR-574-3p	0.32	*GNB1L*	1.11	-0.72	1.50 x 10^{-4}	0.0428	2
hsa-miR-1273h-3p	0.49	*SND1*	1.12	-0.72	1.51 x 10^{-4}	0.0428	4
hsa-miR-9-5p	0.57	*PRKRIP1*	1.11	-0.72	1.51 x 10^{-4}	0.0428	2
hsa-miR-1273h-3p	0.49	*SUPT5H*	1.16	-0.72	1.52 x 10^{-4}	0.0428	1
hsa-miR-320a	0.55	*SEMA4D*	1.23	-0.72	1.53 x 10^{-4}	0.0428	3
hsa-miR-1273h-3p	0.49	*CNN2*	1.28	-0.72	1.54 x 10^{-4}	0.0428	1
hsa-miR-148a-3p	2.23	*HSPA4L*	0.58	-0.72	1.55 x 10^{-4}	0.0428	4
hsa-miR-155-3p	2.27	*KAT2A*	0.77	-0.72	1.55 x 10^{-4}	0.0428	1
hsa-miR-574-3p	0.32	*SLC25A44*	1.22	-0.72	1.55 x 10^{-4}	0.0428	1
hsa-miR-155-3p	2.27	*LBH*	0.52	-0.72	1.57 x 10^{-4}	0.0431	1
hsa-miR-320a	0.55	*PARP15*	1.30	-0.72	1.59 x 10^{-4}	0.0432	1
hsa-miR-574-3p	0.32	*RER1*	1.11	-0.72	1.61 x 10^{-4}	0.0434	1
hsa-miR-1273h-3p	0.49	*VEGFB*	1.41	-0.72	1.61 x 10^{-4}	0.0434	4
hsa-miR-15a-5p	1.62	*HMGN5*	0.78	-0.72	1.62 x 10^{-4}	0.0434	1
hsa-miR-125a-5p	0.16	*C9*	1.13	-0.72	1.63 x 10^{-4}	0.0434	1
hsa-miR-155-3p	2.27	*RHOV*	0.73	-0.72	1.64 x 10^{-4}	0.0434	1
hsa-miR-378a-3p	0.68	*GTF2IRD2B*	1.18	-0.72	1.66 x 10^{-4}	0.0434	1
hsa-miR-320a	0.55	*INHBA*	1.29	-0.72	1.68 x 10^{-4}	0.0435	1
hsa-miR-320a	0.55	*PLSCR3*	1.31	-0.72	1.71 x 10^{-4}	0.0440	4
hsa-miR-155-3p	2.27	*TIMM50*	0.85	-0.72	1.73 x 10^{-4}	0.0440	1
hsa-miR-378a-3p	0.68	*LSP1*	1.48	-0.72	1.74 x 10^{-4}	0.0440	1
hsa-miR-320a	0.55	*PARD6G*	2.01	-0.72	1.74 x 10^{-4}	0.0440	4
hsa-miR-574-3p	0.32	*ALG1*	1.19	-0.72	1.75 x 10^{-4}	0.0440	1
hsa-miR-138-5p	0.32	*ST8SIA2*	1.14	-0.72	1.76 x 10^{-4}	0.0440	3
hsa-miR-574-3p	0.32	*OSTC*	1.13	-0.72	1.77 x 10^{-4}	0.0440	1
hsa-miR-378a-3p	0.68	*CYP1A1*	1.41	-0.72	1.77 x 10^{-4}	0.0440	1
hsa-miR-454-5p	2.14	*DAAM2*	0.62	-0.72	1.78 x 10^{-4}	0.0440	2
hsa-miR-148a-3p	2.23	*PNPT1*	0.78	-0.72	1.78 x 10^{-4}	0.0440	2
hsa-miR-378a-3p	0.68	*TNFAIP1*	1.10	-0.72	1.79 x 10^{-4}	0.0442	2
hsa-miR-155-3p	2.27	*ZFP62*	0.84	-0.72	1.84 x 10^{-4}	0.0446	5
hsa-miR-941	0.54	*PDE1B*	1.28	-0.72	1.85 x 10^{-4}	0.0446	2
hsa-miR-26b-5p	1.78	*GNPDA1*	0.75	-0.72	1.85 x 10^{-4}	0.0446	2
hsa-miR-1273h-3p	0.49	*BCKDK*	1.21	-0.72	1.86 x 10^{-4}	0.0446	2
hsa-miR-378a-3p	0.68	*GABARAP*	1.14	-0.72	1.87 x 10^{-4}	0.0446	1
hsa-miR-9-5p	0.57	*MAPKAPK2*	1.22	-0.72	1.88 x 10^{-4}	0.0446	6
hsa-miR-138-5p	0.32	*CNN2*	1.28	-0.72	1.88 x 10^{-4}	0.0446	1
hsa-miR-345-5p	0.56	*NENF*	1.22	-0.72	1.89 x 10^{-4}	0.0446	2
hsa-miR-155-3p	2.27	*SLC41A1*	0.56	-0.72	1.90 x 10^{-4}	0.0446	1

hsa-miR-155-3p	2.27	*OGDHL*	0.68	-0.72	1.92 x 10^{-4}	0.0446	1
hsa-miR-142-3p	1.47	*MKKS*	0.77	-0.72	1.92 x 10^{-4}	0.0446	1
hsa-miR-378a-3p	0.68	*PARD6G*	2.01	-0.72	1.93 x 10^{-4}	0.0446	2
hsa-miR-125a-5p	0.16	*TCF19*	1.20	-0.72	1.95 x 10^{-4}	0.0447	1
hsa-miR-1273h-3p	0.49	*CYB5D2*	1.24	-0.72	1.95 x 10^{-4}	0.0447	3
hsa-miR-345-5p	0.56	*TOLLIP*	1.16	-0.72	1.95 x 10^{-4}	0.0447	4
hsa-miR-374a-3p	1.74	*GPBP1*	0.81	-0.72	1.97 x 10^{-4}	0.0447	2
hsa-miR-155-3p	2.27	*SIRT5*	0.81	-0.71	1.99 x 10^{-4}	0.0448	1
hsa-miR-374a-3p	1.74	*ZFAT*	0.79	-0.71	2.00 x 10^{-4}	0.0448	3
hsa-miR-26b-5p	1.78	*LDLR*	0.85	-0.71	2.00 x 10^{-4}	0.0448	2
hsa-miR-320a	0.55	*MSRA*	1.48	-0.71	2.03 x 10^{-4}	0.0451	1
hsa-miR-378a-3p	0.68	*TMEM109*	1.32	-0.71	2.07 x 10^{-4}	0.0452	2
hsa-miR-378a-3p	0.68	*EDEM1*	1.25	-0.71	2.08 x 10^{-4}	0.0452	5
hsa-miR-1273h-3p	0.49	*PIGT*	1.13	-0.71	2.08 x 10^{-4}	0.0452	1
hsa-miR-1273h-3p	0.49	*WASF2*	1.14	-0.71	2.09 x 10^{-4}	0.0452	3
hsa-miR-142-3p	1.47	*TBRG4*	0.86	-0.71	2.10 x 10^{-4}	0.0452	1
hsa-miR-15a-5p	1.62	*MTX3*	0.72	-0.71	2.10 x 10^{-4}	0.0452	1
hsa-miR-374a-3p	1.74	*TARBP2*	0.87	-0.71	2.10 x 10^{-4}	0.0452	1
hsa-miR-27a-5p	1.66	*KMO*	1.36	-0.71	2.11 x 10^{-4}	0.0453	2
hsa-let-7d-3p	0.71	*CYB5R1*	1.11	-0.71	2.13 x 10^{-4}	0.0454	1
hsa-miR-320a	0.55	*RBM3*	2.00	-0.71	2.16 x 10^{-4}	0.0459	2
hsa-miR-378a-3p	0.68	*FA2H*	1.18	-0.71	2.17 x 10^{-4}	0.0460	1
hsa-miR-155-3p	2.27	*AEBP2*	0.90	-0.71	2.19 x 10^{-4}	0.0462	3
hsa-miR-138-5p	0.32	*GPS2*	1.13	-0.71	2.20 x 10^{-4}	0.0462	2
hsa-miR-378a-3p	0.68	*GGA3*	1.15	-0.71	2.20 x 10^{-4}	0.0462	1
hsa-miR-1273h-3p	0.49	*UROD*	1.27	-0.71	2.24 x 10^{-4}	0.0464	1
hsa-miR-941	0.54	*MCF2L2*	1.13	-0.71	2.27 x 10^{-4}	0.0466	2
hsa-miR-378a-3p	0.68	*CTNNBIP1*	1.14	-0.71	2.28 x 10^{-4}	0.0466	3
hsa-miR-155-3p	2.27	*TYSND1*	0.86	-0.71	2.28 x 10^{-4}	0.0466	3
hsa-miR-941	0.54	*RIC8A*	1.15	-0.71	2.29 x 10^{-4}	0.0466	2
hsa-miR-378a-3p	0.68	*PSD3*	1.18	-0.71	2.31 x 10^{-4}	0.0468	5
hsa-miR-378a-3p	0.68	*PLCB3*	1.23	-0.71	2.34 x 10^{-4}	0.0468	2
hsa-miR-155-3p	2.27	*RPL31*	0.90	-0.71	2.34 x 10^{-4}	0.0468	1
hsa-miR-345-5p	0.56	*ST8SIA2*	1.14	-0.71	2.34 x 10^{-4}	0.0468	4
hsa-miR-130b-3p	0.69	*CARM1*	1.09	-0.71	2.36 x 10^{-4}	0.0470	2
hsa-miR-22-3p	1.79	*HJURP*	0.85	-0.71	2.37 x 10^{-4}	0.0471	1
hsa-miR-320a	0.55	*MATN2*	1.19	-0.71	2.37 x 10^{-4}	0.0471	2
hsa-miR-425-5p	0.73	*INHBA*	1.29	-0.71	2.40 x 10^{-4}	0.0473	1
hsa-miR-9-5p	0.57	*GPR114*	1.90	-0.71	2.42 x 10^{-4}	0.0474	4
hsa-miR-574-3p	0.32	*UTP3*	1.22	-0.71	2.43 x 10^{-4}	0.0474	1
hsa-miR-320a	0.55	*ARRDC3*	1.52	-0.71	2.43 x 10^{-4}	0.0474	4
hsa-miR-155-3p	2.27	*TRAK1*	0.76	-0.71	2.44 x 10^{-4}	0.0474	5
hsa-miR-378a-3p	0.68	*SLC2A5*	2.62	-0.71	2.45 x 10^{-4}	0.0475	1

hsa-miR-1273h-3p	0.49	*RER1*	1.11	-0.71	2.46 x 10^{-4}	0.0475	1
hsa-miR-574-3p	0.32	*CLN6*	1.21	-0.71	2.49 x 10^{-4}	0.0477	2
hsa-miR-574-3p	0.32	*CDV3*	1.12	-0.71	2.50 x 10^{-4}	0.0477	2
hsa-miR-181d-5p	0.55	*STAT5A*	1.25	-0.71	2.51 x 10^{-4}	0.0477	3
hsa-miR-320a	0.55	*CCL22*	2.56	-0.71	2.52 x 10^{-4}	0.0477	3
hsa-miR-345-5p	0.56	*FSTL3*	1.64	-0.71	2.53 x 10^{-4}	0.0477	1
hsa-miR-378a-3p	0.68	*SEMA4D*	1.23	-0.71	2.57 x 10^{-4}	0.0480	3
hsa-miR-320a	0.55	*UROD*	1.27	-0.71	2.58 x 10^{-4}	0.0481	2
hsa-miR-320a	0.55	*CES2*	1.17	-0.70	2.59 x 10^{-4}	0.0481	2
hsa-miR-345-5p	0.56	*PODXL*	1.14	-0.70	2.63 x 10^{-4}	0.0483	4
hsa-miR-574-3p	0.32	*RBBP9*	1.30	-0.70	2.63 x 10^{-4}	0.0483	2
hsa-miR-138-5p	0.32	*POM121C*	1.15	-0.70	2.64 x 10^{-4}	0.0483	2
hsa-miR-27a-5p	1.66	*SELL*	0.44	-0.70	2.64 x 10^{-4}	0.0483	2
hsa-miR-9-5p	0.57	*INHBA*	1.29	-0.70	2.65 x 10^{-4}	0.0483	2
hsa-miR-15a-5p	1.62	*NARS2*	0.82	-0.70	2.65 x 10^{-4}	0.0483	1
hsa-miR-320a	0.55	*SF3A1*	1.17	-0.70	2.66 x 10^{-4}	0.0483	6
hsa-miR-574-3p	0.32	*SEC61A1*	1.20	-0.70	2.67 x 10^{-4}	0.0483	1
hsa-miR-378a-3p	0.68	*ABCA10*	1.39	-0.70	2.70 x 10^{-4}	0.0483	1
hsa-miR-320a	0.55	*VEGFB*	1.41	-0.70	2.70 x 10^{-4}	0.0483	2
hsa-miR-320a	0.55	*LPXN*	1.47	-0.70	2.71 x 10^{-4}	0.0483	1
hsa-miR-378a-3p	0.68	*NEK8*	1.19	-0.70	2.73 x 10^{-4}	0.0484	1
hsa-miR-320a	0.55	*PIGT*	1.13	-0.70	2.73 x 10^{-4}	0.0484	2
hsa-miR-9-5p	0.57	*DNAH1*	1.18	-0.70	2.73 x 10^{-4}	0.0484	2
hsa-miR-138-5p	0.32	*CCNK*	1.17	-0.70	2.75 x 10^{-4}	0.0484	2
hsa-miR-15a-5p	1.62	*RBBP5*	0.87	-0.70	2.76 x 10^{-4}	0.0484	3
hsa-miR-155-3p	2.27	*IKZF2*	0.68	-0.70	2.77 x 10^{-4}	0.0485	2
hsa-miR-320a	0.55	*BRD9*	1.14	-0.70	2.79 x 10^{-4}	0.0485	2
hsa-miR-574-3p	0.32	*MBD2*	1.11	-0.70	2.81 x 10^{-4}	0.0485	1
hsa-miR-378a-3p	0.68	*YIPF3*	1.35	-0.70	2.82 x 10^{-4}	0.0485	1
hsa-miR-320a	0.55	*ABCA5*	1.37	-0.70	2.82 x 10^{-4}	0.0485	3
hsa-miR-138-5p	0.32	*WBP2*	1.20	-0.70	2.84 x 10^{-4}	0.0487	4
hsa-miR-374a-3p	1.74	*RBM39*	0.90	-0.70	2.87 x 10^{-4}	0.0487	2
hsa-miR-320a	0.55	*STS*	1.86	-0.70	2.90 x 10^{-4}	0.0487	6
hsa-miR-378a-3p	0.68	*GTF2IRD2*	1.17	-0.70	2.91 x 10^{-4}	0.0487	1
hsa-miR-125a-5p	0.16	*MAP4*	1.08	-0.70	2.92 x 10^{-4}	0.0487	4
hsa-miR-378a-3p	0.68	*MARK2*	1.14	-0.70	2.92 x 10^{-4}	0.0487	1
hsa-miR-374a-3p	1.74	*ZC3HC1*	0.88	-0.70	2.92 x 10^{-4}	0.0487	1
hsa-miR-505-3p	0.5	*KIAA0195*	1.10	-0.70	2.92 x 10^{-4}	0.0487	1
hsa-miR-345-5p	0.56	*RAP2C*	1.09	-0.70	2.93 x 10^{-4}	0.0487	2
hsa-miR-148a-3p	2.23	*ZNF184*	0.77	-0.70	2.93 x 10^{-4}	0.0487	1
hsa-miR-138-5p	0.32	*GGA3*	1.15	-0.70	2.93 x 10^{-4}	0.0487	7
hsa-miR-15a-5p	1.62	*TRAK1*	0.76	-0.70	2.94 x 10^{-4}	0.0487	4
hsa-miR-15a-5p	1.62	*PTPN2*	0.84	-0.70	2.98 x 10^{-4}	0.0487	1

hsa-miR-320a	0.55	*BLCAP*	1.18	-0.70	2.98×10^{-4}	0.0487	5
hsa-miR-1273h-3p	0.49	*SF3A2*	1.14	-0.70	2.98×10^{-4}	0.0487	1
hsa-miR-9-5p	0.57	*POLR3C*	1.17	-0.70	3.01×10^{-4}	0.0487	1
hsa-miR-9-5p	0.57	*ZZEF1*	1.10	-0.70	3.01×10^{-4}	0.0487	2
hsa-miR-744-5p	1.59	*CD59*	0.84	-0.70	3.01×10^{-4}	0.0487	2
hsa-miR-345-5p	0.56	*SLC12A4*	1.29	-0.70	3.01×10^{-4}	0.0487	1
hsa-miR-320a	0.55	*FAM129A*	1.70	-0.70	3.02×10^{-4}	0.0487	7
hsa-miR-574-3p	0.32	*POM121*	1.17	-0.70	3.05×10^{-4}	0.0487	2
hsa-miR-320a	0.55	*H6PD*	1.28	-0.70	3.06×10^{-4}	0.0487	3
hsa-miR-320a	0.55	*BCL2L15*	1.19	-0.70	3.06×10^{-4}	0.0487	3
hsa-miR-574-3p	0.32	*LANCL2*	1.20	-0.70	3.06×10^{-4}	0.0487	1
hsa-miR-1273h-3p	0.49	*GPBP1L1*	1.08	-0.70	3.06×10^{-4}	0.0487	4
hsa-miR-15a-5p	1.62	*CENPA*	0.82	-0.70	3.07×10^{-4}	0.0487	2
hsa-miR-194-5p	0.6	*CREM*	1.34	-0.70	3.10×10^{-4}	0.0487	1
hsa-miR-142-3p	1.47	*SLC9A3R1*	0.83	-0.70	3.10×10^{-4}	0.0487	2
hsa-miR-130b-3p	0.69	*B4GALT5*	1.21	-0.70	3.10×10^{-4}	0.0487	7
hsa-miR-320a	0.55	*WBP2*	1.20	-0.70	3.10×10^{-4}	0.0487	2
hsa-miR-148a-3p	2.23	*TBCK*	0.83	-0.70	3.12×10^{-4}	0.0487	1
hsa-miR-941	0.54	*HEXA*	1.22	-0.70	3.12×10^{-4}	0.0487	1
hsa-miR-148b-3p	2.32	*SNX16*	0.65	-0.70	3.13×10^{-4}	0.0487	2
hsa-miR-26b-5p	1.78	*EVI5*	0.65	-0.70	3.14×10^{-4}	0.0487	2
hsa-miR-15a-5p	1.62	*ZMYND11*	0.87	-0.70	3.14×10^{-4}	0.0487	3
hsa-miR-378a-3p	0.68	*SF3A1*	1.17	-0.70	3.15×10^{-4}	0.0487	3
hsa-miR-142-3p	1.47	*MRPS11*	0.83	-0.70	3.17×10^{-4}	0.0487	2
hsa-miR-125a-5p	0.16	*FLG*	1.14	-0.70	3.17×10^{-4}	0.0487	4
hsa-miR-505-3p	0.5	*TSC2*	1.21	-0.70	3.18×10^{-4}	0.0487	1
hsa-miR-1273h-3p	0.49	*TOLLIP*	1.16	-0.70	3.18×10^{-4}	0.0487	2
hsa-miR-505-3p	0.5	*BOD1*	1.11	-0.70	3.20×10^{-4}	0.0487	2
hsa-miR-155-3p	2.27	*DTX4*	0.56	-0.70	3.20×10^{-4}	0.0487	4
hsa-miR-320a	0.55	*MED15*	1.30	-0.70	3.20×10^{-4}	0.0487	2
hsa-miR-374a-3p	1.74	*IL17RB*	0.29	-0.70	3.21×10^{-4}	0.0487	1
hsa-miR-320a	0.55	*SMAP2*	1.14	-0.70	3.23×10^{-4}	0.0489	4
hsa-miR-378a-3p	0.68	*MKNK1*	1.19	-0.70	3.26×10^{-4}	0.0489	2
hsa-miR-320a	0.55	*SND1*	1.12	-0.70	3.27×10^{-4}	0.0489	6
hsa-miR-374a-3p	1.74	*DTX4*	0.56	-0.70	3.27×10^{-4}	0.0489	1
hsa-miR-378a-3p	0.68	*ADRM1*	1.25	-0.70	3.28×10^{-4}	0.0489	1
hsa-miR-574-3p	0.32	*PRDM1*	1.45	-0.70	3.28×10^{-4}	0.0489	1
hsa-miR-26b-5p	1.78	*MED14*	0.91	-0.70	3.29×10^{-4}	0.0489	1
hsa-miR-155-3p	2.27	*RNF114*	0.81	-0.70	3.31×10^{-4}	0.0491	6
hsa-miR-374a-3p	1.74	*SSTR2*	0.60	-0.70	3.33×10^{-4}	0.0492	2
hsa-miR-15a-5p	1.62	*ZBTB11*	0.86	-0.70	3.34×10^{-4}	0.0492	3
hsa-miR-574-3p	0.32	*P2RY1*	1.20	-0.69	3.40×10^{-4}	0.0496	2
hsa-miR-155-3p	2.27	*PIAS1*	0.90	-0.69	3.40×10^{-4}	0.0496	3

hsa-miR-155-3p	2.27	*MIF*	0.85	-0.69	3.43×10^{-4}	0.0497	1
hsa-miR-1273h-3p	0.49	*ZZEF1*	1.10	-0.69	3.43×10^{-4}	0.0497	4
hsa-miR-9-5p	0.57	*NSD1*	1.12	-0.69	3.46×10^{-4}	0.0499	3

Correlations between miRNAs and mRNAs differentially expressed between lithium excellent responders and non-responders were analyzed using miRComb. Multiple testing correction was performed according to BH. The *in-silico* score was calculated by summing the number of algorithms predicting each miRNA-mRNA pair (seven prediction algorithms were tested: MiRWalk, MicroT v4, MiRanda, miRDB, RNA22, RNAhybrid and TargetScan). Abbreviations: FC, fold change; FDR, false discovery rate.

Table S3. Pairs of miRNAs and mRNAs differentially expressed after *in vitro* lithium treatment exclusively in excellent responders, negatively correlated and predicted by in-silico algorithms.

miRNA	FC	mRNA	FC	Pearson's r	*p*	FDR *q*	*In-silico* score
hsa-miR-374a-5p	1.41	*B4GALT6*	0.82	-0.59	0.0041	0.35	5
hsa-miR-29b-3p	1.18	*ZNF577*	0.88	-0.49	0.0162	0.65	5
hsa-miR-29b-3p	1.54	*RFX7*	0.92	-0.49	0.0164	0.65	6
hsa-miR-106b-5p	1.41	*ZNF493*	0.84	-0.47	0.0216	0.67	2
hsa-miR-23a-3p	1.28	*FFAR2*	0.84	-0.42	0.0363	0.86	1
hsa-miR-27a-3p	1.25	*ZNF493*	0.84	-0.41	0.0413	0.86	6
hsa-miR-27a-3p	1.18	*IKBIP*	0.87	-0.40	0.0459	0.86	1

Correlations between miRNAs and mRNAs differentially expressed after in vitro lithium treatment exclusively in excellent responders were analyzed using miRComb. No correlation was significant after multiple testing correction according to BH. The *in-silico* score was calculated by summing the number of algorithms predicting each miRNA-mRNA pair (seven prediction algorithms were tested: MiRWalk, MicroT v4, MiRanda, miRDB, RNA22, RNAhybrid and TargetScan). Abbreviations: FC, fold change; FDR, false discovery rate.

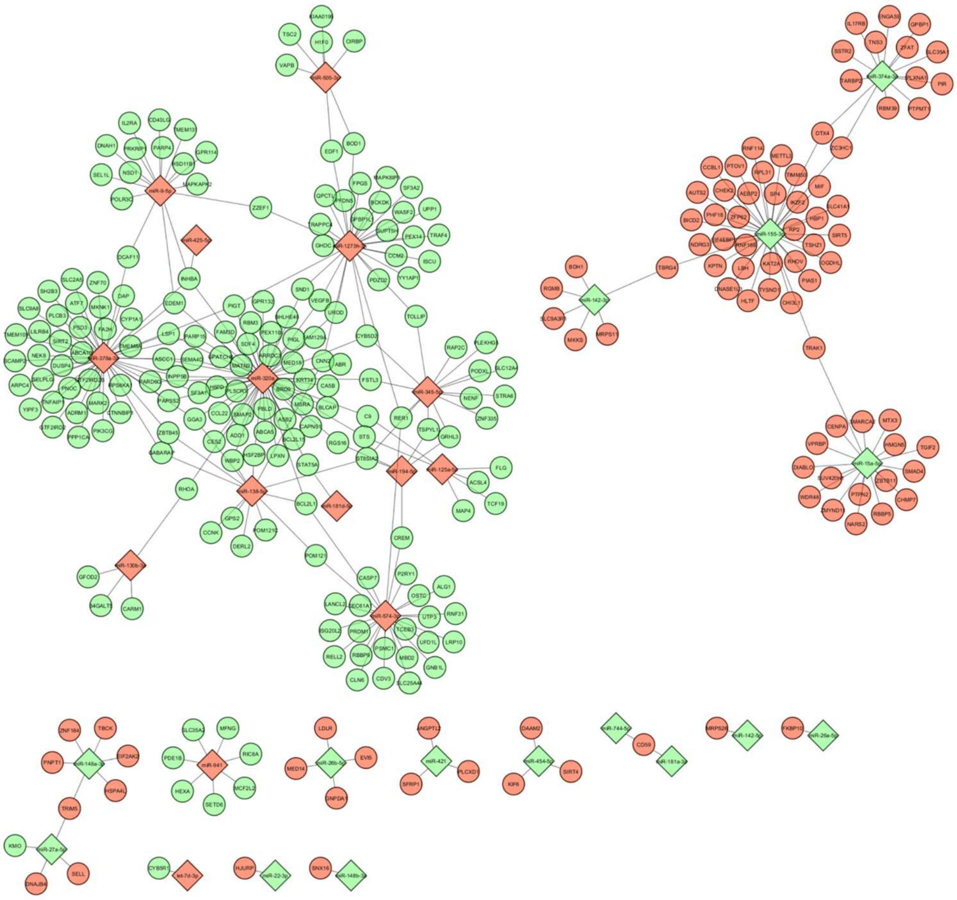

Figure S1. Interaction network between miRNAs and mRNAs differentially expressed between lithium excellent responders and non-responders. The interaction network of miRNAs and mRNAs differentially expressed between lithium excellent responders and non-responders was obtained using a force-directed layout algorithm in Cytoscape. miRNAs are indicated with a diamond, target mRNAs with circles. Down-regulated miRNAs or mRNAs are shown in red, while up-regulated miRNAs or mRNAs are shown in green. The network shows two main cluster of down-regulated and up-regulated miRNAs centered around miR-320a and miR-155-3p, respectively.

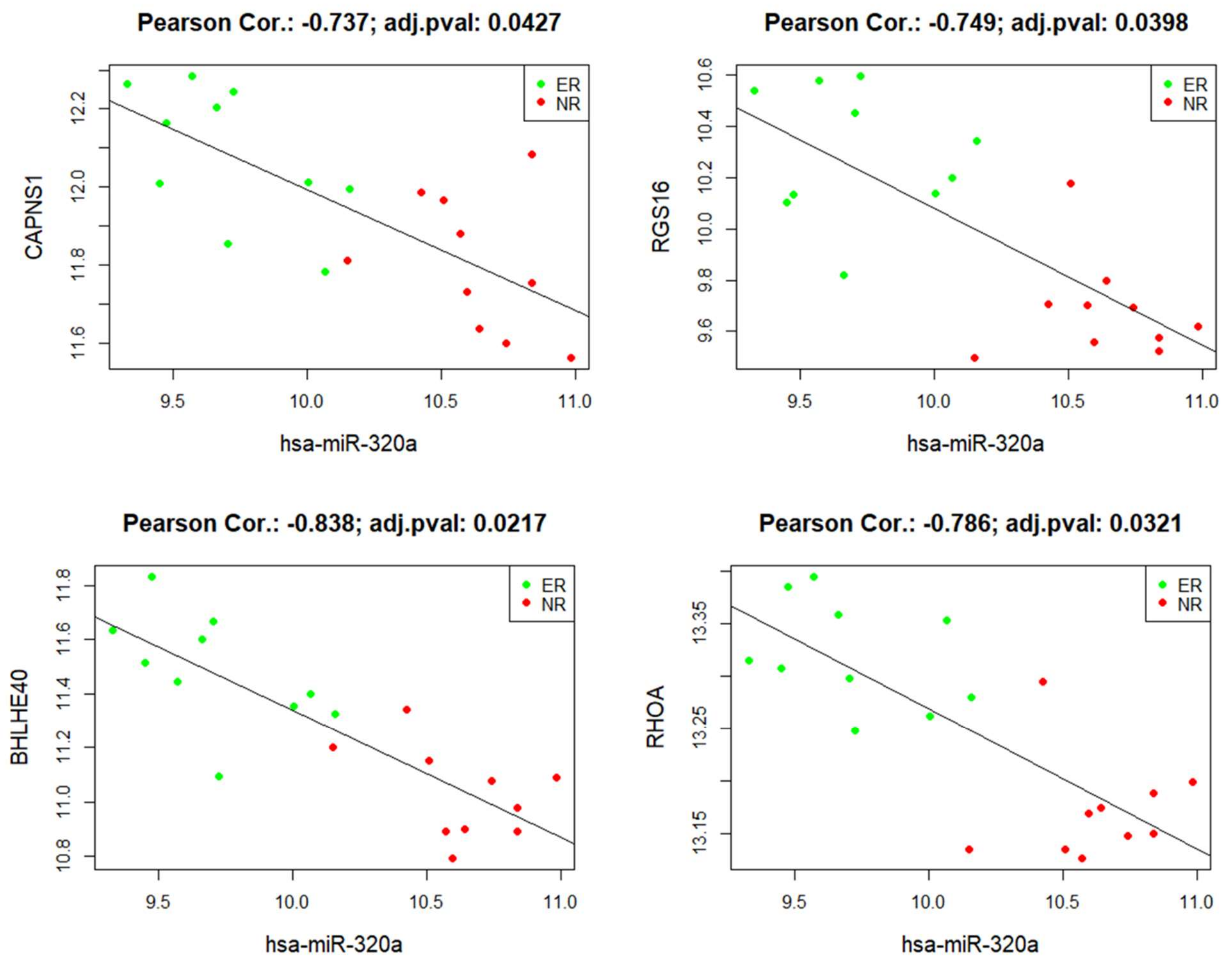

Figure S2. Correlations between the expression levels of hsa-miR-320a and its targets *CAPNS1*, *RGS16*, *BHLHE40* and *RHOA* in lithium excellent responders and non-responders. Abbreviations: adj, adjusted; cor, correlation; ER, excellent responders; NR, non-responders.

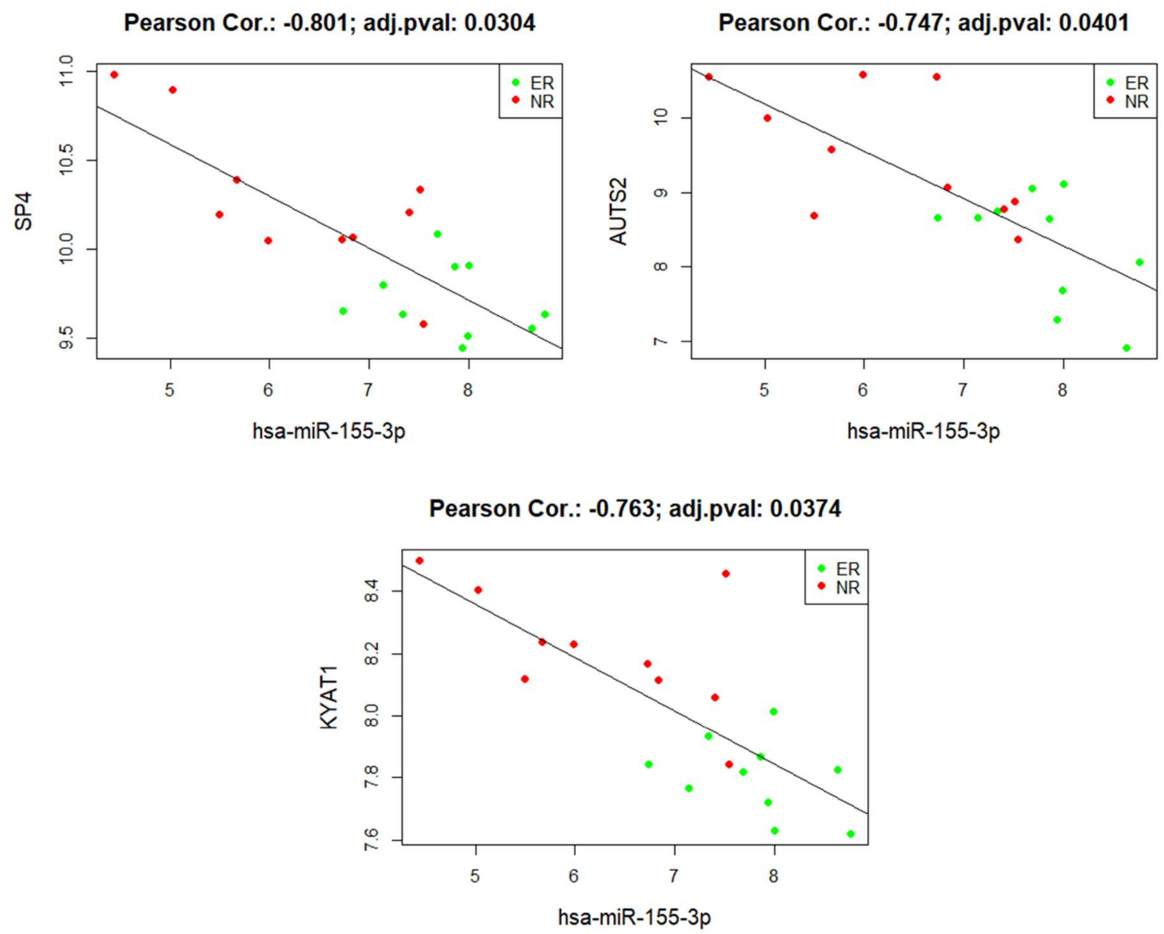

Figure S3. Correlations between the expression levels of hsa-miR-155-3p and its targets *SP4*, *AUTS2* and *KYAT1* in lithium excellent responders and non-responders. Abbreviations: adj, adjusted; cor, correlation; ER, excellent responders; NR, non-responders.
